# More Frequent On-Site Dialysis May Hasten Return to Home for Nursing Home Patients with End-Stage Kidney Disease

**DOI:** 10.34067/KID.0000000000000487

**Published:** 2024-06-07

**Authors:** Eran Y. Bellin, Alice M. Hellebrand, William T. Markis, Jordan G. Ledvina, Steven M. Kaplan, Nathan W. Levin, Allen M. Kaufman

**Affiliations:** 1 Departments of Epidemiology and Population Health and Medicine, Albert Einstein College of Medicine, Bronx, New York; 2 Dialyze Direct, Neptune City, New Jersey; 3 WTM Consulting, Lakewood Ranch, Florida; 4 Internal Medicine, Mount Sinai Icahn School of Medicine, New York, New York

**Keywords:** chronic dialysis, epidemiology and outcomes, hospitalization, outcomes, renal dialysis, survival

## Abstract

**Key Points:**

- Prior efficacy study—established that more frequent dialysis achieved better outcomes than CONVENTIONAL dialysis in outpatients.
- We undertook an effectiveness observational on-site nursing home study (*N*=195) comparing on-site more frequent dialysis with CONVENTIONAL dialysis.
- More frequent dialysis patients, despite being sicker at baseline, returned home faster than CONVENTIONALLY dialyzed patients without worsened death or hospitalization.

**Background:**

A direct outcome comparison between skilled nursing facility (SNF) patients receiving on-site more frequent dialysis (MFD) targeting 14 hours of treatment over five sessions weekly compared with on-site CONVENTIONAL dialysis for death, hospitalization, and speed of returning home has not been reported.

**Methods:**

From January 1, 2022, to July 1, 2023, in a retrospective prospective observational design, using an intention-to-treat and competing risk strategy, all new admissions for an on-site SNF dialysis service done to nursing homes with on-site MFD were compared with admissions to nursing homes providing on-site CONVENTIONAL dialysis for the outcome goal of 90-day cumulative incidence of discharge to home, while monitoring safety issues represented by the competing risks of hospitalization and death.

**Results:**

In total, 10,246 MFD dialytic episodes and 3451 CONVENTIONAL dialytic episodes were studied in 195 nursing homes in 12 states. At baseline, the MFD population was consistently sicker than CONVENTIONAL dialysis population with a first systolic BP of <100 mm Hg in 13% versus 7.6% (*P <* 0.001), lower mean hemoglobin (9.3 versus 10.4 g/dl; *P* < 0.001), lower iron saturation (25.7% versus 26.6%; *P* = 0.02), higher Charlson score (3.5 versus 3.0; *P* < 0.001), higher mean age (67.6 versus 66.7; *P* < 0.001), more complicated diabetes (31% versus 24%; *P* < 0.001), cerebrovascular disease (12.6% versus 6.8%; *P*<0.001), and congestive heart failure (24% versus 18%). At 42 days, discharge to home was 25% greater in the MFD than CONVENTIONAL dialysis group (17.5% versus 14%) without worsened hospitalization or death.

**Conclusions:**

Despite a handicap of sicker patients at baseline, real-world application of MFD appears to hasten return to home from SNFs compared with CONVENTIONAL dialysis. The findings suggest that MFD allows for SNF acceptance of sicker patients, presumably permitting earlier discharge from hospital, without safety compromise as measured by death or rehospitalization, benefitting hospitals, patients, and payers.

## Visual Abstract

**Figure:**
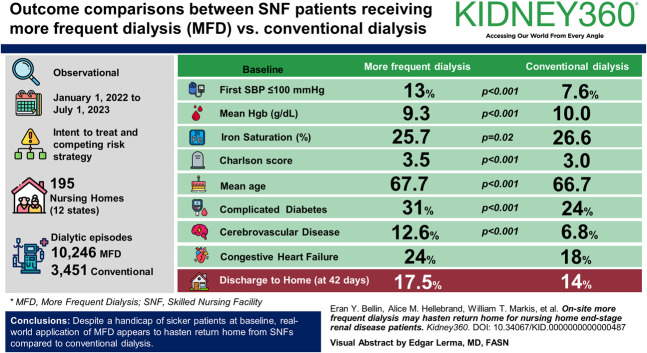

In our previous research, we described on-site more frequent dialysis (MFD) in skilled nursing facilities (SNFs) within a national service provider.^1^ These on-site MFD patients experienced a more rapid, patient-reported, postdialysis recovery time (DRT) compared with the published experience of non-SNF populations.^2^ Among residents participating in SNF rehabilitation programs, these on-site MFD patients also demonstrated superior participation in tphysical therapy compared with recipients of off-site CONVENTIONAL dialysis and equivalent physical therapy participation to that of the general, nondialysis, SNF population.^3^ Although these observations were consistent with previous impressions of the utility of MFD,^4–9^ a critical health services question remained: Does in-SNF MFD improve hard outcomes compared with in-SNF CONVENTIONAL 3×/wk dialysis?

There are primarily three competing outcomes of an in-SNF dialysis patient's nursing home stay—discharge to home, hospitalization, and death, with early discharge to home as the most favorable outcome. We asked whether in-SNF MFD hastens discharge to home.

Our previous interest in comparing MFD with CONVENTIONAL dialysis was stymied by the lack of dialysis records and outcomes data for an in-SNF on-site conventionally treated control group. Over the past 1.5 years, Dialyze Direct has absorbed organizations delivering in-SNF on-site dialysis three times per week, which gave us access to the relevant control group for this direct comparison.

## Methods

### Setting

Patients with ESKD residing in SNFs received on-site dialysis services in 195 nursing homes across 12 states with one of two modalities: >3×/wk more frequent dialysis (MFD) and 3×/wk dialysis (CONVENTIONAL). MFD prescription targets five times a week with a minimum of 14 hours of treatment per week and a weekly stdKt/V ≥2.0 provided *via* NxStage System One technology pursuant to a physician’s order. CONVENTIONAL dialysis prescribes a target frequency of 3×/wk with a minimum of 10.5 hours of treatment per week and weekly stdKt/V ≥2.0. Care was provided by Dialyze Direct, as described previously.^1^ A specialized and regionally coordinated staff operated on-site in the SNF to deliver treatments. In the past 1.5 years, additional nursing home sites with on-site CONVENTIONAL (3×/wk) dialysis programs were absorbed.

Dialysis care data are collected and stored by Dialyze Direct within Clarity (Visonex, Green Bay, WI) and fed into a central data repository used for Quality Improvement and Institutional Review Board-approved research.

This study adhered to the principles outlined in the Declaration of Helsinki. The Institutional Review Board (WCG Institutional Review Board, Puyallup, WA) ruled this study protocol to be exempt under 45 CFR § 46.104(d)(4) of the Common Rule with a full waiver of health insurance portability and accountability act authorization for use and disclosure of protected health information for this research.

### Subject Constructs

#### Dialytic Episode

The dialytic episode is defined as the interval starting from the patient’s first in-SNF dialysis session, inclusive of all subsequent dialysis sessions, until treatment cessation at that facility due to hospitalization, death, transfer to another facility, transfer to home, or withdrawal from therapy. On readmission to the SNF, the first dialysis session after readmission initiates a new dialytic episode. We excluded from the competing risk analysis all the dialysis experiences of those patients with dialytic episode outcomes recorded as “transferred,” “recovered,” or “withdrawn,” retaining only those who went home, were discharged from hospital, died, or were administratively censored by the study's end.

#### Post-DRT

Beginning November 4, 2019, Dialyze Direct initiated a company-wide effort to collect DRT information. At each dialysis session, the patient was asked the duration of recovery time to baseline function after their antecedent dialysis session with specific categorical answer choices: 0–½ hour, ½–1 hour, 1–2 hours, 2–4 hours, 4–8 hours, 8–12 hours, by next morning, or not even by next morning. Nurses recorded whether the patient was unable to respond due to cognitive impairment or physical inability to speak, including intubation. The <2-hour cut point for “rapid DRT” was chosen to match previously published experience in this population.^2^

#### Descriptive Statistics

Stata 18 (College Station, TX) was used for data management, descriptive statistics, mixed model logistic regression, Kaplan–Meier survival analysis provided as its complement cumulative discharge proportion, and competing risk survival analysis. Categorical variable proportions were compared with the chi-square test. Continuous variables of two groups were compared with the *t* test and Kruskal–Wallis nonparametric test.

The race/ethnicity variable was built sequentially by first assigning ethnicity and then designating the non-Hispanic population as White, Black, or other/unknown.

Charlson scores and diagnoses were ascertained in the year antecedent to SNF admission using Stata and the method of Ludvigsson,^10^ building on the earlier work of Quan.^11^

Laboratory test values used for group comparisons were restricted to the first laboratory test drawn within 7 days before to 7 days after the dialytic episode start date to assure that these values represent the baseline state of the patient at time of admission and not a consequence of the specific modality of in-SNF dialysis care.

To analyze multiple DRT reports within a single dialytic episode, a mixed model logistic regression used a separate constant random effect at the dialytic episode level (utilizing melogit and then meqrlogit if melogit failed). The predetermined explanatory variable “dialysis style” (MFD versus CONVENTIONAL) was assessed for its ability to predict report of rapid DRT (<2 hours)—the outcome variable chosen in advance of model building, consistent with our previous work.^2^

To describe the “as-treated” dialysis intensity of the two dialysis methodologies, we evaluated individual pseudoweeks, which consisted of consecutive 7-day windows within a dialytic episode anchored on the first dialysis received in the first full week of the said episode. We excluded any episodes with SNF stay ≤1 week, the first week of dialysis experience for those episode duration greater than 1 week, and the final week of dialysis within a nursing home stay. This approach limits the pseudoweek method to patients with a clear, potentially influenceable outcome and a guaranteed minimum full 7 days of care. It eliminates the confounding effects of timing of last dialysis in the pre-SNF admission or incomplete treatment week at the end of the dialytic episode. Within each pseudoweek, we can determine the count of dialysis sessions, the cumulative time for dialysis, and the cumulative net fluid removed to describe the as-treated experience of those with intention-to-treat MFD or CONVENTIONAL dialysis.

The net fluid removed is obtained by subtracting the postweight from preweight of each dialysis session. The ultrafiltration rate (UFR) is calculated using this net fluid removed divided by dialysis time in hours per kg of postdialysis weight (ml/h per kilogram).

#### Dialysis Treatment Variable—Intention to Treat

Dialyze Direct provides CONVENTIONAL dialysis and MFD at different in-SNF sites with characteristically distinct equipment. While, theoretically, three times per week dialysis could be performed with NxStage equipment, this would require ≥5 hours of treatment duration per dialysis session, which is impractical and never attempted. Treatment assignment for this study follows intention-to-treat analysis, independent of actual received treatment frequency. This method is essential to prevent a reverse causality bias where sicker patients who more often decline therapy would become classified as receiving treatment at CONVENTIONAL dialysis frequency although they were on the MFD platform. By utilizing the intention-to-treat paradigm, real-world delivered dialysis frequency affected by patient choice and clinical deterioration does not induce inappropriate patient group reassignment.

#### Competing Risk Statistical Outcome Method

Classic competing risk analysis uses the competing risk method of Fine and Gray,^12–17^ modeling the cumulative incidence (subdistribution hazard) or risk of the competing risks over an appropriate time period to develop an answer to the relevant health services question: Should MFD or CONVENTIONAL dialysis be chosen for dialysis patients recently discharged from a hospital to a SNF?

The weakness of this method in its usual simplest execution (as in stcrreg in Stata^18^ [College Station, TX]) is that it assumes proportionality of subdistribution hazard to permit a single (time-invariant) ratio with which to judge the effect of the variable's two values (in our case MFD versus CONVENTIONAL dialysis). Frequently, this assumption of time-invariant proportionality is violated,^19^ and many questionable intellectual contortions are required to extract meaning, relying on low-power tests to neurotically assuage “biostatistical guilt.” In addition, the classic method makes no attempt to define the cumulative incidence outcomes of the two populations (MFD and CONVENTIONAL dialysis), no effort to measure its relative subdistribution ratio change over time (as this is axiomatically forbidden), and no effort to document the absolute risk difference with evolving time. Fortunately, Mozumder^20–22^ and colleagues developed a statistical routine in Stata (stpm2cr) that includes flexible cubic splines with five degrees of freedom to model baseline cumulative incidence, permits modeling the interaction of time with three degrees of freedom (allowing the subdistribution hazard ratio to modify over time), and can generate meaningful visualization of cumulative incidence difference between the two dialysis styles. This method enables demonstration of cumulative risk changes longitudinally (an important issue discussed below). We set out to build models of three cumulative risk functions (home, hospitalization, and death) and compare a classic model assuming proportionality (#1), a model assuming time dependency in the subdistribution hazard ratio between MFD and CONVENTIONAL dialysis in the outcomes, home and hospitalization (#2), and a model assuming time dependency in the three outcomes (#3). The best model will be chosen using the Akaike information criteria^23–26^ that capture the amount of information lost by the model and penalize model choice by model complexity to reduce modeling of noise. The lower the Akaike score, the better.

The end point in this analysis does not suffer from the usual perverse problem of multiple undesirable competing risks. When all competing risks are undesirable, a specific risk can look better if an increase in another undesirable outcome precludes the incidence of that risk. For example, in the case of competing risks after hospital discharge, if readmission or death were the only two outcomes, then as death increased, readmission cumulative incidence would decline. Obviously, a decline in readmissions due to a death increase cannot be interpreted as desirable. Fortunately, our target of interest, “going home,” is a positive outcome, so an increase in returning home as a consequence of a decrease in death or hospitalization is desirable. If death or hospitalization incidence increases, then our end point of interest “going home” would decrease. Hence, our end point of interest behaves as a reliable indicator of good health outcome—a measure whose increase would be indisputably desirable.

## Results

From January 1, 2022, to July 1, 2023, 10,246 MFD dialytic episodes and 3451 CONVENTIONAL dialytic episodes were initiated. Table 1 provides demographics, laboratory values, Charlson scores, diagnoses proportions, and a summary of the first systolic BP before the first dialysis in the nursing home stay. To summarize, the SNFs using an MFD-style dialysis accepted consistently sicker dialysis patients than SNFs providing the CONVENTIONAL dialysis population with a first systolic BP ≤100 mm Hg in 13% versus 7.6% (*P* < 0.001), lower mean hemoglobin (9.3 versus 10.4 g/dl; *P* < 0.001), lower iron saturation (25.7% versus 26.6%; *P* = 0.02), higher Charlson score (3.5 versus 3.0; *P* < 0.001), and higher mean age (67.6 versus 66.7; *P* < 0.001). Driving the Charlson score difference are higher prevalences of serious medical conditions in MFD versus CONVENTIONAL dialysis: complicated diabetes (31% versus 24%; *P* < 0.001), cerebrovascular disease (12.6% versus 6.8%; *P* < 0.001), and congestive heart failure (24% versus 18%) (Table 1).

**Table 1:** Baseline patient characteristics upon nursing home admission repeated for each dialytic episode by style of dialysis—more frequent dialysis versus CONVENTIONAL dialysis

Variable	*P* Value	MFD (*N*=10,246)		CONVENTIONAL Dialysis (*N*=3451)	
		*N* (%)	Mean (SD)	*N* (%)	Mean (SD)
Age	*P* = 0.000	10,246	67.7 (12.1)	3451	66.7 (12.4)
**Sex**	*P* = 0.27				
Male			4808 (47%)		1582 (46%)
Female			5438 (53%)		1869 (54%)
**Race/ethnicity**	*P* < 0.001				
White			3345 (34%)		989 (29%)
Hispanic			745 (7%)		142 (4%)
Black			2962 (29%)		1064 (31%)
Other/unknown			3104 (30%)		1257 (36%)
**Access (primary)**	*P* = 0.09				
Catheter			5544 (62%)		1892 (64%)
Either arteriovenous fistula or AV graft			3357 (38%)		1062 (36%)
First systolic BP before first dialysis	*P* < 0.001		129.3 (27)		137.3 (27.9)
**First systolic BP (mm Hg)**	*P* < 0.001				
≤100			1342 (13%)		262 (7.6%)
>100			8904 (87%)		3189 (92%)
Hemoglobin (g/dl)	*P* < 0.001	*N*=6378	9.3 (2.2)	*N*=1961	10 (4.1)
Iron saturation (%)	*P* = 0.022	*N*=6032	25.7%(14.3)	*N*=1838	26.6 (14.8)
Ferritin (ng/ml)	*P* = 0.225	*N*=6734	956 (821)	*N*=2181	760 (16.3)
Parathyroid hormone (pg/ml)	*P* = 0.33	*N*=6087	364 (487)	*N*=2198	376 (498)
Phosphorous (mg/dl)	*P* = 0.06	*N*=6161	4.8 (1.6)	*N*=1894	4.7 (1.6)
Calcium (mg/dl)	*P* = 0.041	*N*=6756	8.7 (0.86)	*N*=2123	8.7 (0.82)
Albumin (g/dl)	*P* = 0.99	*N*=5974	3.09 (0.53)	*N*=1885	3.09 (0.54)
Calcium corrected (mg/dl)	*P* = 0.006	*N*=6054	9.5 (0.79)	*N*=1883	9.497 (0.2)
Charlson score	*P* < 0.001	*N*=9532	3.5 (1.82)	*N*=3019	3.05 (1.6)
**Charlson categories (%)**					
Acute MI	*P* < 0.001		310 (3%)		45 (1.3%)
CHF	*P* < 0.001		2329 (24%)		543 (18%)
PVD	*P*<0.001		840 (8.8%)		145 (4.8%)
Cerebrovascular disease	*P* < 0.001		1197 (12.6%)		205 (6.8%)
COPD	*P* = 0.001		1105 (11.6%)		283 (9.4%)
Chronic other pulmonary	*P* < 0.001		310 (3.3%)		61 (2%)
Dementia	*P* = 0.014		352 (3.7%)		83 (2.8%)
Hemi or paraplegia	*P* = 0.553		147 (1.5%)		42 (1.4%)
Diabetes uncomplicated	*P* = 0.102		59 (0.6%)		11 (0.3%)
Diabetes complicated	*P* < 0.001		2918 (31%)		722 (24%)
Renal disease	*P* = 0.001		9343 (98%)		2927 (97%)
Mild liver disease	*P* < 0.001		295 (3.1%)		46 (1.5%)
Liver mod or severe disease	*P* = 0.886		30 (0.3%)		9 (0.3%)
Peptic ulcer disease	*P* = 0.151		86 (0.9%)		19 (0.6%)
Cancer	*P* = 0.001		297 (3.1%)		59 (2%)
Metastatic cancer	*P* = 0.019		12 (0.13%)		10 (0.33%)
AIDS	*P* < 0.001		714 (7%)		432 (12.6%)
No. of SNF			149 (76%)		46 (24%)
No. of states			12		6
No. of unique patients			5504 (75%)		1829 (25%)

AV, arteriovenous; CHF, congestive heart failure; COPD, chronic obstructive pulmonary disease; MFD, more frequent dialysis; MI, myocardial infarction; PVD, peripheral vascular disease; SNF, skilled nursing facility.

Of all 13,697 dialytic episodes, 941 were excluded from the competing risk outcome analysis because their disposition by June 3, 2023, was either transferred (site unknown), withdrawn from dialysis, or recovered, leaving 12,756 (93%) dialytic episodes available for modeling.

### Dialysis Description

Using the pseudoweek methodology, described above, of the eligible 163,030 dialyses (127,993 MFD; 79%), we create 43,166 pseudoweeks (30,943 MFD; 72%) drawn from 6286 distinct dialytic episodes (4694 MFD; 75%). A summary of descriptive values are provided in Tables 2 and 3.^27–31^

**Table 2:** Dialysis session characteristics by dialysis style (pseudoweek methodology)

Item Described	MFD		CONVENTIONAL Dialysis		*P* Value
	Mean	25th, 50th, 75th Percentile	Mean	25th, 50th, 75th Percentile	
Number of dialyses per week	4.1	4, 4, 5	2.9	3, 3, 3	<0.001
Dialysis time (hr/wk)	11.7	10, 12.2, 14.2	9.7	8.6, 10, 11.1	<0.001
Net fluid removed per session (L)	1.06	0.5, 1, 1.5	1.56	0.8, 1.5, 2.3	<0.001
UFR per session (ml/hr per kilogram)	4.5	2.3, 4.5, 6.8	5.6	3.3, 5.6. 8.4	<0.001
spKt/V^a^	0.68	0.57, 0.66, 0.77	1.45	1.17, 1.42, 1.67	<0.001

MFD, more frequent dialysis; UFR, ultrafiltration rate.

a Single dialysis measured Kt/V.

**Table 3:** stdKt/V achieved using single pool urea Kt/V (cutoff provided) by actual in measurement week dialysis count in CONVENTIONAL and more frequent dialysis nursing home patients (pseudoweek method)

Dialysis Type and Frequency	% Achieve stdKt/V≥2 (Using Cutoff spKt/V^a^)	% Achieve stdKt/V≥2 (2.4) (Using Cutoff spKt/V)
**CONVENTIONAL dialysis (*N*=2855)**		
3×/wk *N*=22,345 (81%)	76% (1.2)	82% (1.14)
**MFD (*N*=8565)**		
3×/wk *N*=1159 (14%)	4% (1.14)	<1% (1.17)
4×/wk *N*=2969 (35%)	45% (0.72)	9% (0.96)
5×/wk *N*=4050 (47%)	81% (0.54)	37% (0.68)

MFD, more frequent dialysis.

a Threshold values provided in NxStage Home Therapy manual.^27–31^

In total, 120,613 (94%) of 127,993 MFD dialysis sessions reported a short recovery time (<2 hours) compared with 31,853 (91%) of the 35,037 CONVENTIONAL dialysis sessions (chi-square; *P* < 0.001). This was confirmed by the mixed model odds ratio of 8.2 (confidence interval [CI] [5.9 to 11.4]; *P* < 0.001).

### Cumulative Incidence by Competing Risk Predicted by Dialysis Style

Using the technique of Mozumder with Akaike information criteria, the best model both for 0–14 days and for 0–90 days has a time-varying subdistribution hazard ratio for the variable dialysis style with outcomes of home and hospital but not death (Table 4).

**Table 4:** AIC of three models

Model	AIC (0–14 d)	AIC (0–90 d)
#1 style proportional	31,862.855	57,632.299
#2 tvc home and hospital	31,858.106^a^	56,725.756^a^
#3 tvc home, hospital, death	31,862.572	56,729.99

tvc is time varying ratio for subdistribution hazard more frequent dialysis versus CONVENTIONAL dialysis for outcome specified.

a Best model by Akaike information criteria.

### Cumulative Incidence of Discharge to Home, Hospital, or Death (Days 0–90)

**Figure 1:**
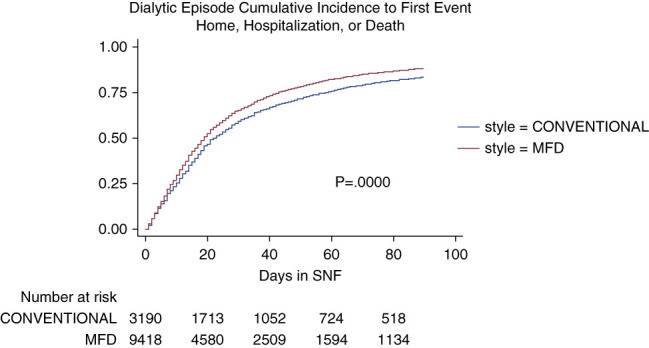
**Dialytic episodes cumulative incidence to first event (all cause: discharge home, hospital, or death).** MFD, more frequent dialysis.

Cumulative incidence of any first event of discharge to home, hospitalization, or death in the first 90 days of a dialytic episode is summarized in Figure 1. The risk table below Figure 1 indicates the number of dialysis patients still in the nursing home on that day and still capable of testifying to future cumulative incidence of the three outcomes. Subsequent 90-day windows (90–180, 180–270, 270–360) for dialytic episodes with patient numbers at the start of an interval are provided in the Supplement (Supplemental Figures 5–16).

**Figure 2:**
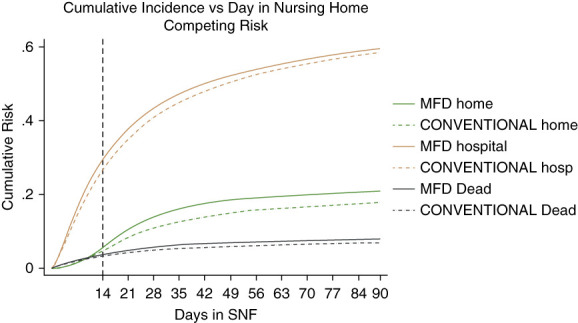
Dialytic episodes competing risk cumulative incidence discharge home, hospital, death, MFD versus CONVENTIONAL.

**Figure 3:**
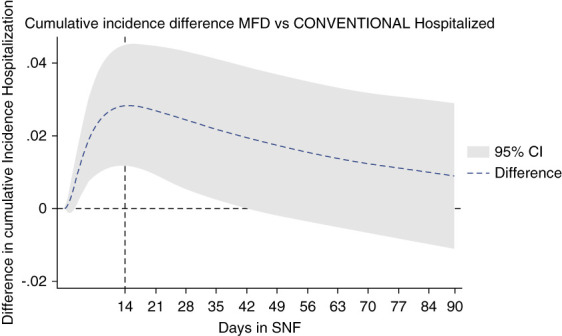
Dialytic episodes cumulative incidence difference discharge to hospital, MFD versus CONVENTIONAL.

**Figure 4:**
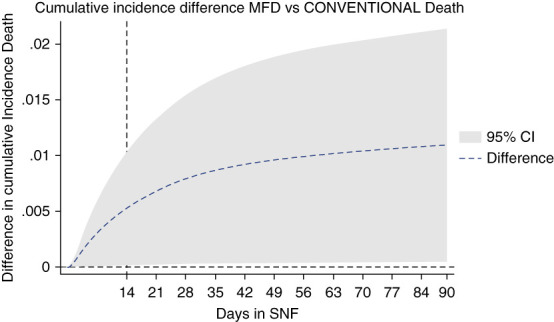
Dialytic episodes cumulative incidence difference death, MFD versus CONVENTIONAL.

**Figure 5:**
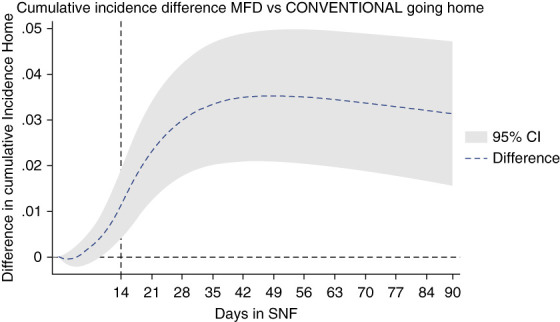
Dialytic episodes cumulative incidence difference discharged home, MFD versus CONVENTIONAL.

Figure 2 shows the breakdown of cumulative incidence from Figure 1, displaying cumulative competing risks of discharge to home, hospitalization, or death. In Figures 3–5, cumulative incidence differences between MFD and CONVENTIONAL dialysis for the competing outcomes of hospitalization, death, and home are shown. Graphic display of the 95% CI permits assessment of persistence of difference or whether zero is included (null hypothesis).

MFD risk of hospitalization was worse in the first 14 days (Figure 3) which suggests a sicker MFD group at baseline before any outcome could be reasonably attributed to dialysis style. Over the ensuing follow-up (days 14–90), the baseline handicap is overcome with the difference reduced to nonsignificance (CI overlapping zero).

The CI of the difference in death risk hovers close to zero, likely a consequence of the relatively low death rate (Figure 4).

What is most striking is the risk of going home (Figure 5). After 14 days, it is very clear from the graph that MFD is superior to CONVENTIONAL dialysis in rapid return to home. At 42 days, discharge to home was 25% greater in the MFD than CONVENTIONAL dialysis group (17.5% versus 14%). The difference is statistically significant, unambiguous, and persistent.

**Figure 6:**
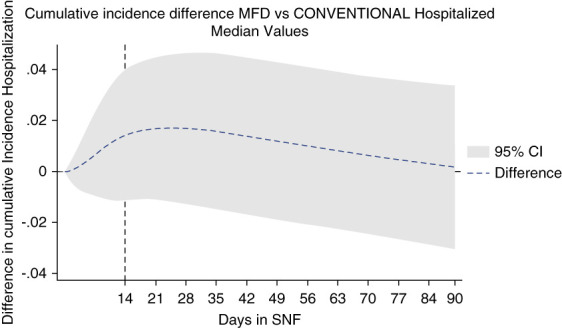
Dialytic episodes cumulative incidence difference hospitalized, MFD versus CONVENTIONAL in adjusted model evaluated at median covariate values.

In a sensitivity analysis, we adjusted for baseline differences in age, Charlson score, hemoglobin, iron saturation, dichotomized systolic BP before first dialysis, and primary access using a subcohort with available data for input as covariates in the model without requiring significance for inclusion. In this subpopulation, the proportion with primary catheter access is 2612/4296 (61%) for MFD and 807/1295 (62%) for CONVENTIONAL dialysis (*P* = 0.33). Time to first combined event for this subset of dialytic episodes with all variables available is provided with a risk table (Supplemental Figure 1). Forcing these variables into the adjusted competing risk model, we then display the cumulative incidence difference evaluated at the median values of the population of Charlson score (3), hemoglobin (9), iron saturation (22.5), age (68), systolic BP >100 mm Hg at baseline, and primary access intravenous catheter. The adjusted model at the median values displays the same dramatic improvement in return to home in the MFD group (Supplemental Figure 2 and 3) and a clear elimination of any significant difference between MFD and CONVENTIONAL dialysis in hospitalization cumulative incidence (even within the first 14 days) (Figure 6) or in death (Supplemental Figure 4), where both respective CIs include zero.

## Discussion—Analytic Strategy

We used two strategies to draw inferences from our observations. The first was to evaluate the unadjusted data where the MFD group was consistently disadvantaged at baseline in those variables thought to be important for health status—hemoglobin, iron saturation, age, prefirst dialysis systolic BP, Charlson score, and differential prevalences of clinically important conditions driving the Charlson score (severe diabetes, cerebrovascular disease, and congestive heart failure). We utilized a competing risk-modeling process to compare cumulative incidence rates and their differences, which demonstrated a dramatic, consistently faster discharge to home in the MFD group. The early 0–14 days increase in hospitalization is likely reflective of baseline differences and cannot reasonably be attributed to the short exposure to a particular dialysis style. The fact that this difference attenuates as MFD has time to exert its influence and subsequently the CI quickly crosses zero supports our suspicion that the initial increase is due to baseline differences. Death cumulative incidence difference at its 95% CI hovers at zero. Despite a clear baseline disadvantage, return to home was hastened with MFD without a safety signal from death or hospitalization.

A secondary analysis attempted to account for these variables of baseline disadvantage as covariates, reviewing the cumulative incidence differences at their population median values. The results were even more striking. Home discharge was hastened in MFD and now adjusted for baseline inequalities, hospitalization and death CIs both comfortably included zero, even within the first 14 days.

Why should MFD hasten discharge to home? Average weekly dialysis time in MFD was 21% greater than in CONVENTIONAL dialysis patients (11.7/9.7 hours), consistent with that observed in the landmark Frequent Hemodialysis Network (FHN) Daily Trial^9^ comparing six times a week dialysis with thrice weekly (12.7/10.4 hours). The landmark study demonstrated statistically significant difference using both coprimary combined end point of death and left ventricular mass or death and decrease in the physical health composite score. Since urea is a nontoxin, it has long been suspected that another variable “time on dialysis” has independent outcome importance because of its correlation with removal of sodium, volume, and middle molecules.^32,33^ The randomized, controlled, HEMO study^34^ demonstrated that increasing removal of urea through increased dose or increased membrane flux in thrice weekly dialysis failed to achieve benefit, disproving the singular importance of urea clearance expected from previous work.^35^ The failure of increased dialysis time in FHN Nocturnal Trial (six times a week) to improve outcome has been attributed to its absurdly underpowered size (*N*=87 patients).^36^ Even the landmark efficacy Daily Trial (*N*=245 patients) was acknowledged to be underpowered for a single primary end point such as death or hospitalization and was only salvaged by a prespecified combined outcome. Our effectiveness study, by contrast, while observational, enrolled 5504 MFD patients and 1829 CONVENTIONAL dialysis patients in a posthospitalization nursing home setting and survives the bias against the hypothesis as sicker MFD patients still return home quicker even in the unadjusted analysis.

The average per-treatment measured spKt/v in MFD is dramatically lower than that in CONVENTIONAL diaylsis (0.68 versus 1.45), thereby mitigating urea shifts per treatment and reinforcing the notion that MFD is gentler with less dysequilibrium.^37–39^ Because Raimann *et al.*^40^ have demonstrated a clear relationship between UFR and mortality, the lower UFR in the MFD group (4.5 ml/h per kilogram versus 5.6) may also be an important factor influencing early discharge to home.

We note that the reported rapid post-DRTs (<2 hours) in both groups are remarkably good—91% in CONVENTIONAL dialysis and 94% in MFD. The small reported difference might reflect a more significant underlying difference that permits more effective rehabilitation for early discharge to home.

Two other observations bear emphasis for those planning to study the nursing home population. Both the MFD and CONVENTIONAL dialysis groups in our nursing home population had a small per-session net fluid removal (1.06 versus 1.56 L) compared with that seen in the landmark Daily study of outpatients (2.12 versus 3.06 L), probably due to the functional fluid and oral intake restriction in the nursing home population that does not have easy access to either.

We suspect that the MFD, as actually delivered (average 4.1 times weekly), is not optimal with 25% receiving ≤10 hours weekly (despite the intended 14 hours). The CONVENTIONAL dialysis group, as actually delivered, received ≤8.6 hours weekly in 25%. MFD time delivered was on average 84% (11.7/14 hours) of goal, while CONVENTIONAL dialysis time delivered was on average 93% (9.7/10.5 hours) of goal. We acknowledge the limitation that we cannot say with certainty to what extent failure to achieve time goal affected the relative success of the two modalities. However, it appears that although CONVENTIONAL dialysis was more successful in achieving its time goal, it still failed to better MFD results.

Using Table 3, we can calculate the percent that achieved stdKt/V ≥2 in MFD as 54% (0.12 × 0.04 + 0.45 × 0.35 + 0.81 × 0.47) and in CONVENTIONAL dialysis as 62% (0.81 × 0.76), suggesting that by urea criteria, there is room for improvement although it has been suspected that the method underestimates true stdKt/V. Nonperfect adherence to the desired treatment goal (Table 3) is no surprise as even in the landmark FHN Daily study, only 78% of the patients assigned to undergo hemodialysis six times per week attended at least 80% of the prescribed hemodialysis, which means that 62% achieved the goal.^9^

### Limitations

Using observational data to “prove” causality is challenging. The dramatic difference in Charlson scores reinforces the notion that the MFD population was inherently unhealthier. We cannot exclude the possibility that the newly acquired CONVENTIONAL dialysis practices had not developed the documentation norms of the vintage MFD practices, artificially depressing the CONVENTIONAL Charlson scores. However, unbiased laboratory test evidence is consistent with the impression of a sicker MFD population.

We acknowledge that different nursing homes might have different propensities to discharge early that are independent of the clinical status of the patient and that this is a possible confounding factor unaddressed by our analysis. There is no available validated nursing home outcome metric that can be used as an adjuster. The best approach would be to perform the same competing risk analysis on the nondialysis patients to determine whether the differences we observed are unique to the dialysis population and their respective styles and not observed in the nondialysis SNF patient controls.

In addition, we do not have access to potential adjuster data such as duration or cause of ESKD usually reported in CMS Form 2728. We hope that the large sample size of nursing homes included (149 MFD and 46 CONVENTIONAL dialysis) will average out unmeasured confounders.

The dialytic episode rather than the individual patient is the unit of analysis, and we do not consider the issue of multiple sequential readmissions in the individual patient. The statistical analysis permitting the critical implications of competing risk demands that one of the three outcomes be the terminating event, and does not permit analysis of repeating sequential events. To obtain the critical implications of three competing outcomes, we cannot consider serial readmissions beyond the possible first at the end of the dialytic episode.

### Future Work

The proper assessment of the relationship between dialysis style and mortality and hospitalization requires study of the biologic plausibility of these findings. This requires careful review of each hospital referral with a thorough discharge summary from the hospital upon return and follow-up of all mortality with formalized review. Active surveillance with prospective data collection of dialysis and SNF nurse clinical impressions at transfer to hospital or death should be implemented. Lastly, baseline levels of self-care and mobility collected by SNFs to report in the Minimum Data Set reporting to Centers for Medicare & Medicaid Services within the first 3 days of admission are important to establish comparability of populations. Only through transinstitutional coordination/collaboration (hospital, nursing home, dialysis provider), not yet the national norm nor expectation, can specific preventable issues be identified and addressed.

Confounding of the “dialysis style-outcome” relationship by a “nursing home's usual practice” can only be assessed using clinical information and outcomes on the nondialysis population of each nursing home—a possibility in future collaborative efforts.

Despite being handicapped by sicker patients at baseline, MFD appeared to hasten return to home compared with CONVENTIONAL dialysis. If the implications of this observational study are borne out, it suggests that MFD in the SNF in coordination with hospital information transfer allows for acceptance of sicker patients into the SNF, presumably permitting earlier hospital discharge, while also hastening SNF discharge to home. This benefits hospitals, patients, and payers and provides a model for constructive collaborative efforts in the future.

## Supplementary Material

## Data Availability

Data cannot be shared. Proprietary records of the company used for quality improvement purposes only.

## Supplemental Material

Additional covariate adjusted analyses figures at median values.

90-180 days.

Supplemental Figure 1. Dialytic episodes with available covariates cumulative incidence to first event (all cause: discharge home, hospital, or death) (prevalent patients day 90).

Supplemental Figure 2. Dialytic episodes competing risk cumulative incidence home, hospital, death, MFD versus CONVENTIONAL in adjusted model evaluated at median covariate values (prevalent patients day 90).

Supplemental Figure 3. Dialytic episodes competing risk cumulative incidence difference discharged home, MFD versus CONVENTIONAL in adjusted model evaluated at median covariate values (prevalent patients day 90).

Supplemental Figure 4. Dialytic episodes competing risk cumulative incidence difference hospitalized, MFD versus CONVENTIONAL in adjusted model evaluated at median covariate values (prevalent patients day 90).

Supplemental Figure 5. Dialytic episodes competing risk cumulative incidence difference death, MFD versus CONVENTIONAL in adjusted model evaluated at median covariate values (prevalent patients day 90).

180-270 days.

Supplemental Figure 6. Dialytic episodes with available covariates cumulative incidence to first event (all cause: discharge home, hospital, or death) (prevalent patients day 180).

Supplemental Figure 7. Dialytic episodes competing risk cumulative incidence home, hospital, death, MFD versus CONVENTIONAL in adjusted model evaluated at median covariate values (prevalent patients day 180).

Supplemental Figure 8. Dialytic episodes competing risk cumulative incidence difference discharged home, MFD versus CONVENTIONAL in adjusted model evaluated at median covariate values (prevalent patients day 180).

Supplemental Figure 9. Dialytic episodes competing risk cumulative incidence difference hospitalized, MFD versus CONVENTIONAL in adjusted model evaluated at median covariate values (prevalent patients day 180).

Supplemental Figure 10. Dialytic episodes competing risk cumulative incidence difference death, MFD versus CONVENTIONAL in adjusted model evaluated at median covariate values (prevalent patients day 180).

Supplemental Figure 11. Dialytic episodes with available covariates cumulative incidence to first event (all cause: discharge home, hospital, or death) (prevalent patients day 270).

Supplemental Figure 12. Dialytic episodes with available covariates cumulative incidence to first event (all cause: discharge home, hospital, or death) (prevalent patients day 180) followed through day 360.

Supplemental Figure 13. Dialytic episodes competing risk cumulative incidence home, hospital, death, MFD versus CONVENTIONAL in adjusted model evaluated at median covariate values (prevalent patients day 180) followed through day 360.

Supplemental Figure 14. Dialytic episodes competing risk cumulative incidence difference discharged home, MFD versus CONVENTIONAL in adjusted model evaluated at median covariate values (prevalent patients day 180) followed through day 360.

Supplemental Figure 15. Dialytic episodes competing risk cumulative incidence difference hospitalized, MFD versus CONVENTIONAL in adjusted model evaluated at median covariate values (prevalent patients day 180) followed through day 360.

Supplemental Figure 16. Dialytic episodes competing risk cumulative incidence difference death, MFD versus CONVENTIONAL in adjusted model evaluated at median covariate values (prevalent patients day 180) followed through day 360.
