# Supplementary material for: More Frequent On-Site Dialysis May Hasten Return to Home for Nursing Home Patients with End-Stage Kidney Disease
Source: Kidney360. 2024 Jun 7;5(8):1126–36. doi: 10.34067/KID.0000000000000487 (PMC11371347; doi:10.34067/KID.0000000000000487)
Supplement: Supplementary file 1 [file kidney360-5-1126-s001.pdf]

ASN American Society of Nephrology
JASN Journal of the American Society of Nephrology
KIDNEY360 Accessing Our World From Every Angle
CJASN Clinical Journal of the American Society of Nephrology

ASN Journal Disclosure Form

As per ASN journal policy, I have disclosed any financial relationships or commitments I have held in the past 36 months as included below. I have listed my Current Employer below to indicate there is a relationship requiring disclosure. If no relationship exists, my Current Employer is not listed.

E. Bellin reports the following:
Employer: ; Dialyze Direct; Consultancy: Dialyze Direct; Research Funding: Dialyze Direct; and Other Interests or Relationships: Dialyze Direct for whom I served as senior epidemiologist consultant.

I understand that the information above will be published within the journal article, if accepted, and that failure to comply and/or to accurately and completely report the potential financial conflicts of interest could lead to the following: 1) Prior to publication, article rejection, or 2) Post-publication, sanctions ranging from, but not limited to, issuing a correction, reporting the inaccurate information to the authors' institution, banning authors from submitting work to ASN journals for varying lengths of time, and/or retraction of the published work.

Name: Eran Y. Bellin
Manuscript ID: K360-2024-000232R2
Manuscript Title: "On-site more frequent dialysis may hasten return home for nursing home end-stage renal disease patients
Date of Completion: May 12, 2024
Disclosure Updated Date: May 12, 2024

ASN American Society of Nephrology | JASN Journal of the American Society of Nephrology | KIDNEY360 Accessing Our World From Every Angle | CJASN Clinical Journal of the American Society of Nephrology

ASN Journal Disclosure Form

As per ASN journal policy, I have disclosed any financial relationships or commitments I have held in the past 36 months as included below. I have listed my Current Employer below to indicate there is a relationship requiring disclosure. If no relationship exists, my Current Employer is not listed.

A. Hellebrand reports the following:
Employer: Dialyze Direct; Ownership Interest: Stock options/Dialyze Direct; Advisory or Leadership Role: Strive Health; and Other Interests or Relationships: Member ANNA; Member RHA.

I understand that the information above will be published within the journal article, if accepted, and that failure to comply and/or to accurately and completely report the potential financial conflicts of interest could lead to the following: 1) Prior to publication, article rejection, or 2) Post-publication, sanctions ranging from, but not limited to, issuing a correction, reporting the inaccurate information to the authors' institution, banning authors from submitting work to ASN journals for varying lengths of time, and/or retraction of the published work.

Name: Alice Hellebrand
Manuscript ID: K360-2024-000232R2
Manuscript Title: On-site more frequent dialysis may hasten return home for nursing home end-stage renal disease patients
Date of Completion: May 20, 2024
Disclosure Updated Date: May 20, 2024

ASN American Society of Nephrology | JASN Journal of the American Society of Nephrology | KIDNEY360 Accessing Our World From Every Angle | CJASN Clinical Journal of the American Society of Nephrology

ASN Journal Disclosure Form

As per ASN journal policy, I have disclosed any financial relationships or commitments I have held in the past 36 months as included below. I have listed my Current Employer below to indicate there is a relationship requiring disclosure. If no relationship exists, my Current Employer is not listed.

S. Kaplan reports the following:
Employer: Dialyze Direct

I understand that the information above will be published within the journal article, if accepted, and that failure to comply and/or to accurately and completely report the potential financial conflicts of interest could lead to the following: 1) Prior to publication, article rejection, or 2) Post-publication, sanctions ranging from, but not limited to, issuing a correction, reporting the inaccurate information to the authors' institution, banning authors from submitting work to ASN journals for varying lengths of time, and/or retraction of the published work.

Name: Steven M. Kaplan
Manuscript ID: K360-2024-000232R2
Manuscript Title: On-site more frequent dialysis may hasten return home for nursing home end-stage renal disease patients
Date of Completion: May 22, 2024
Disclosure Updated Date: May 22, 2024

ASN American Society of Nephrology | JASN Journal of the American Society of Nephrology | KIDNEY360 Accessing Our World From Every Angle | CJASN Clinical Journal of the American Society of Nephrology

ASN Journal Disclosure Form

As per ASN journal policy, I have disclosed any financial relationships or commitments I have held in the past 36 months as included below. I have listed my Current Employer below to indicate there is a relationship requiring disclosure. If no relationship exists, my Current Employer is not listed.

J. Ledvina reports the following:
Employer: Dialyze Direct; and Ownership Interest: Dialyze Direct.

I understand that the information above will be published within the journal article, if accepted, and that failure to comply and/or to accurately and completely report the potential financial conflicts of interest could lead to the following: 1) Prior to publication, article rejection, or 2) Post-publication, sanctions ranging from, but not limited to, issuing a correction, reporting the inaccurate information to the authors' institution, banning authors from submitting work to ASN journals for varying lengths of time, and/or retraction of the published work.

Name: Jordan Ledvina
Manuscript ID: K360-2024-000232R2
Manuscript Title: On-site more frequent dialysis may hasten return home for nursing home end-stage renal disease patients
Date of Completion: May 23, 2024
Disclosure Updated Date: May 23, 2024

ASN
American Society of Nephrology
JASN
Journal of the American Society of Nephrology
KIDNEY360
Accessing Our World From Every Angle
CJASN
Clinical Journal of the American Society of Nephrology

ASN Journal Disclosure Form

As per ASN journal policy, I have disclosed any financial relationships or commitments I have held in the past 36 months as included below. I have listed my Current Employer below to indicate there is a relationship requiring disclosure. If no relationship exists, my Current Employer is not listed.

N. Levin reports the following:
Consultancy: Dialyze Direct; Ownership Interest: Fresenius; and Advisory or Leadership Role: Easy Water for Everyone NGO.

I understand that the information above will be published within the journal article, if accepted, and that failure to comply and/or to accurately and completely report the potential financial conflicts of interest could lead to the following: 1) Prior to publication, article rejection, or 2) Post-publication, sanctions ranging from, but not limited to, issuing a correction, reporting the inaccurate information to the authors' institution, banning authors from submitting work to ASN journals for varying lengths of time, and/or retraction of the published work.

Name: Nathan W. Levin
Manuscript ID: K360-2024-000232R1
Manuscript Title: On-site more frequent dialysis may hasten return home for nursing home end-stage renal disease patients
Date of Completion: May 23, 2024
Disclosure Updated Date: May 23, 2024

ASN
American Society of Nephrology
JASN
Journal of the American Society of Nephrology
KIDNEY360
Accessing Our World From Every Angle
CJASN
Clinical Journal of the American Society of Nephrology

ASN Journal Disclosure Form

As per ASN journal policy, I have disclosed any financial relationships or commitments I have held in the past 36 months as included below. I have listed my Current Employer below to indicate there is a relationship requiring disclosure. If no relationship exists, my Current Employer is not listed.

W. Markis reports the following:
Consultancy: Dialyze Direct; and Ownership Interest: No individual stocks in any companies related or relevant to the research conducted for or described in this paper.

I understand that the information above will be published within the journal article, if accepted, and that failure to comply and/or to accurately and completely report the potential financial conflicts of interest could lead to the following: 1) Prior to publication, article rejection, or 2) Post-publication, sanctions ranging from, but not limited to, issuing a correction, reporting the inaccurate information to the authors' institution, banning authors from submitting work to ASN journals for varying lengths of time, and/or retraction of the published work.

Name: William Markis
Manuscript ID: K360-2024-000232R2
Manuscript Title: On-site more frequent dialysis may hasten return home for nursing home end-stage renal disease patients
Date of Completion: May 14, 2024
Disclosure Updated Date: May 14, 2024

ASN American Society of Nephrology | JASN Journal of the American Society of Nephrology | KIDNEY360 Accessing Our World From Every Angle | CJASN Clinical Journal of the American Society of Nephrology

ASN Journal Disclosure Form

As per ASN journal policy, I have disclosed any financial relationships or commitments I have held in the past 36 months as included below. I have listed my Current Employer below to indicate there is a relationship requiring disclosure. If no relationship exists, my Current Employer is not listed.

A. Kaufman reports the following:
Employer: Dialyze Direct; and Ownership Interest: Dialyze Direct.

I understand that the information above will be published within the journal article, if accepted, and that failure to comply and/or to accurately and completely report the potential financial conflicts of interest could lead to the following: 1) Prior to publication, article rejection, or 2) Post-publication, sanctions ranging from, but not limited to, issuing a correction, reporting the inaccurate information to the authors' institution, banning authors from submitting work to ASN journals for varying lengths of time, and/or retraction of the published work.

Name: Allen Kaufman
Manuscript ID: K360-2024-000232R2
Manuscript Title: On-site more frequent dialysis may hasten return home for nursing home end-stage renal disease patients
Date of Completion: May 14, 2024
Disclosure Updated Date: May 14, 2024
