# Supplementary material for: More Frequent On-Site Dialysis May Hasten Return to Home for Nursing Home Patients with End-Stage Kidney Disease
Source: Kidney360. 2024 Jun 7;5(8):1126–36. doi: 10.34067/KID.0000000000000487 (PMC11371347; doi:10.34067/KID.0000000000000487)
Supplement: Supplementary file 2 [file kidney360-5-1126-s002.pdf]

Supplement

In this supplement we replicate the analysis provided in the paper for the first 90 days of a dialytic episode, for consecutive 90 day intervals for the patients who remain at interval start. We first report the complement of the Kaplan Meier survival (cumulative incident to first event) where the event is any first, be it hospitalization, death, or discharge to home (all cause). We provide under the graph the risk table which reveals the number of dialytic episodes with patients who remain in the cohort to experience the temporal downstream competing outcomes.

We then use the same spline choices for degrees of freedom for both dialysis type and outcome interaction with time used in the first 90 day window to model the competing risk experience (MFD vs Conventional) of those prevalent at interval start over the next 90 days using that windows experience to define the coefficients.

Since the last analysis time window we considered (days 270-360) did not initiate with the software possibly due to relatively small sample sizes, we analyzed a 180 days window in the same way beginning at day 180 to complete a view of 360 days in SNF.

The resultant graphs permit the reader to draw conclusions comparing the competing risk outcomes in subsequent time windows after the one we report. We, of course, provide our impressions as well.

Contents

90-180 Days 2

Observation/Impression days 90-180 5

180-270 Days 6

Observation/Impression days 280-270 10

270-360 can not be done with spline modeling 11

180-360 Days 12

Observation/Impression days 180-365 16

90-180 Days

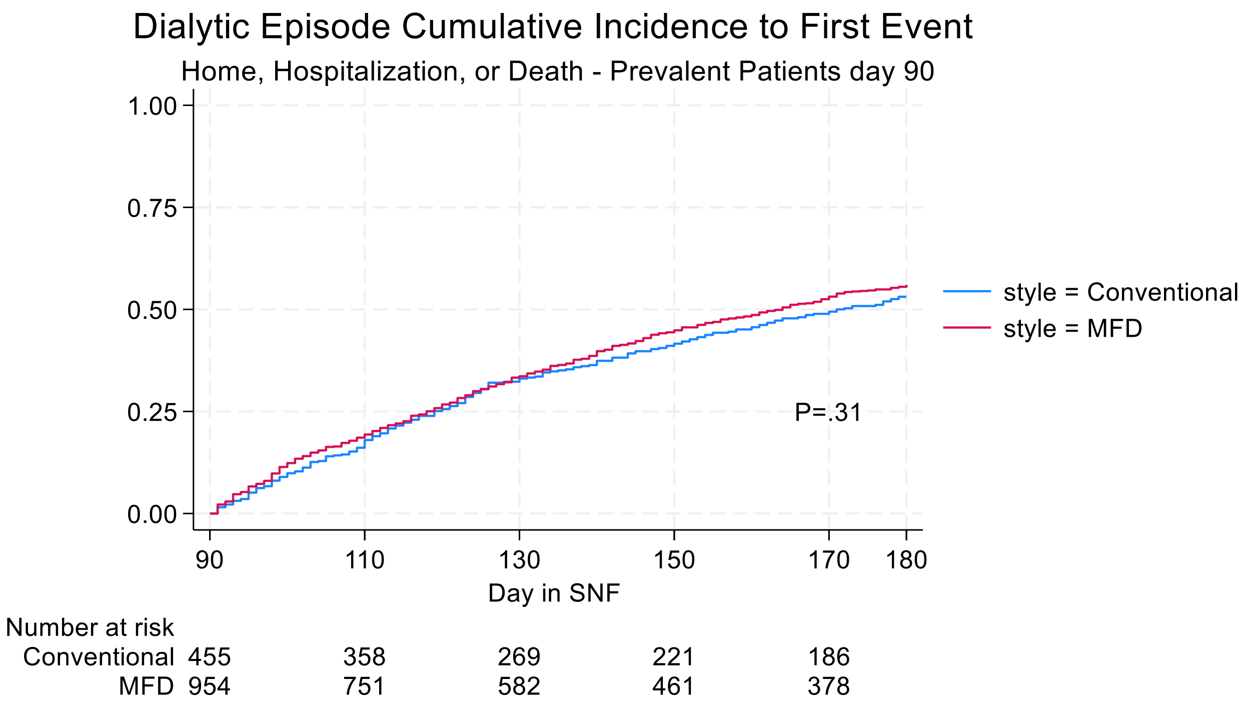

Figure s1. Dialytic Episodes with available covariates Cumulative Incidence to First Event (all cause: discharge home, hospital, or death) (Prevalent Patients Day 90)

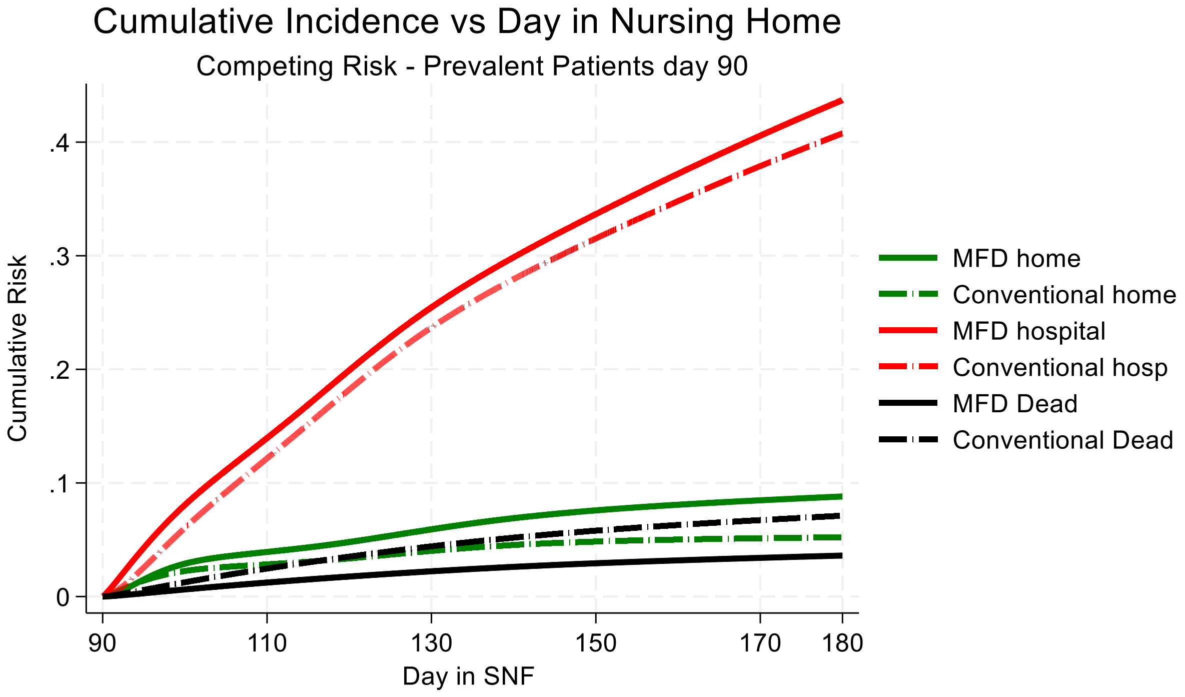

Figure s2. Dialytic Episodes Competing Risk Cumulative incidence home, hospital, death, MFD vs Conventional in adjusted model evaluated at median covariate values (Prevalent patients day 90)

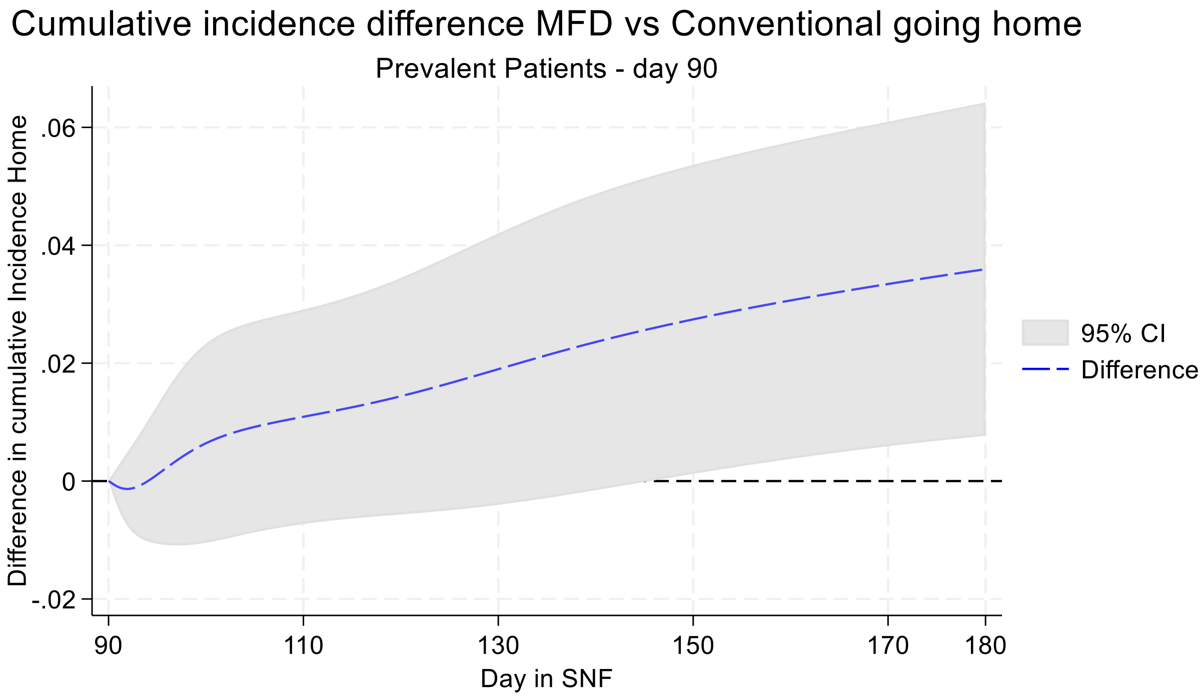

Figure s3. Dialytic Episodes Competing Risk Cumulative Incidence difference discharged home, MFD vs Conventional in adjusted model evaluated at median covariate values (Prevalent patients Day 90)

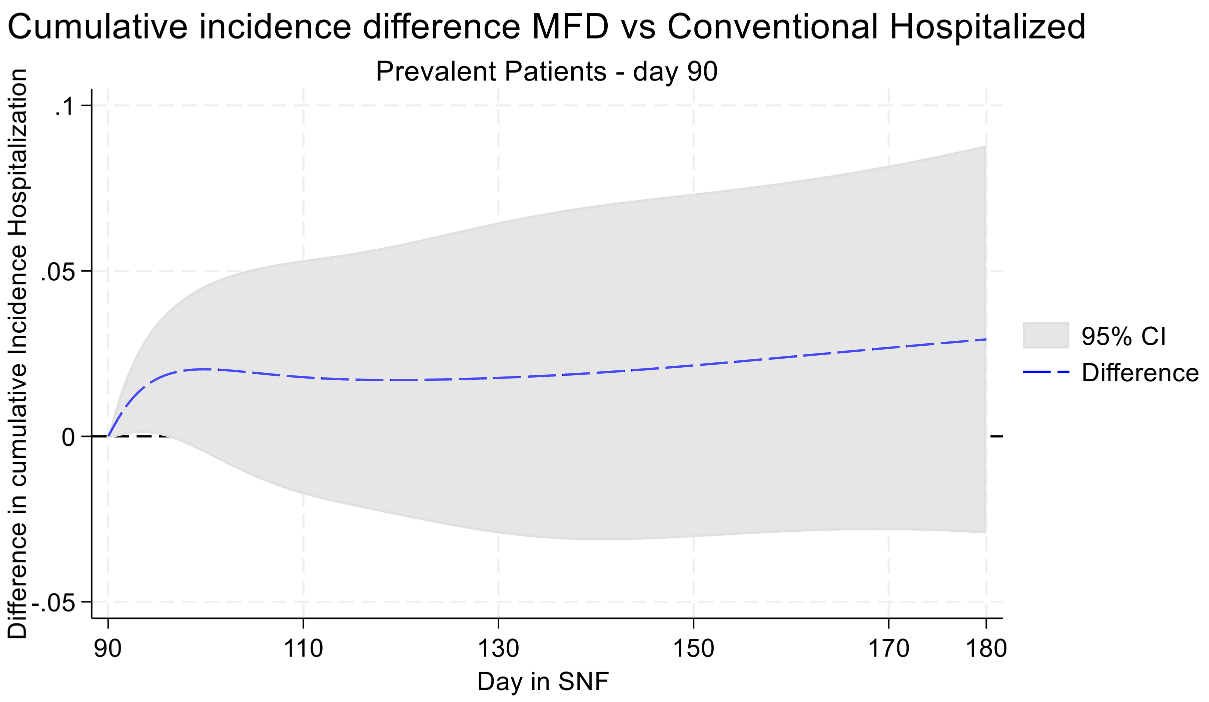

Figure s4. Dialytic Episodes Competing Risk Cumulative Incidence difference Hospitalized, MFD vs Conventional in adjusted model evaluated at median covariate values (Prevalent patients Day 90)

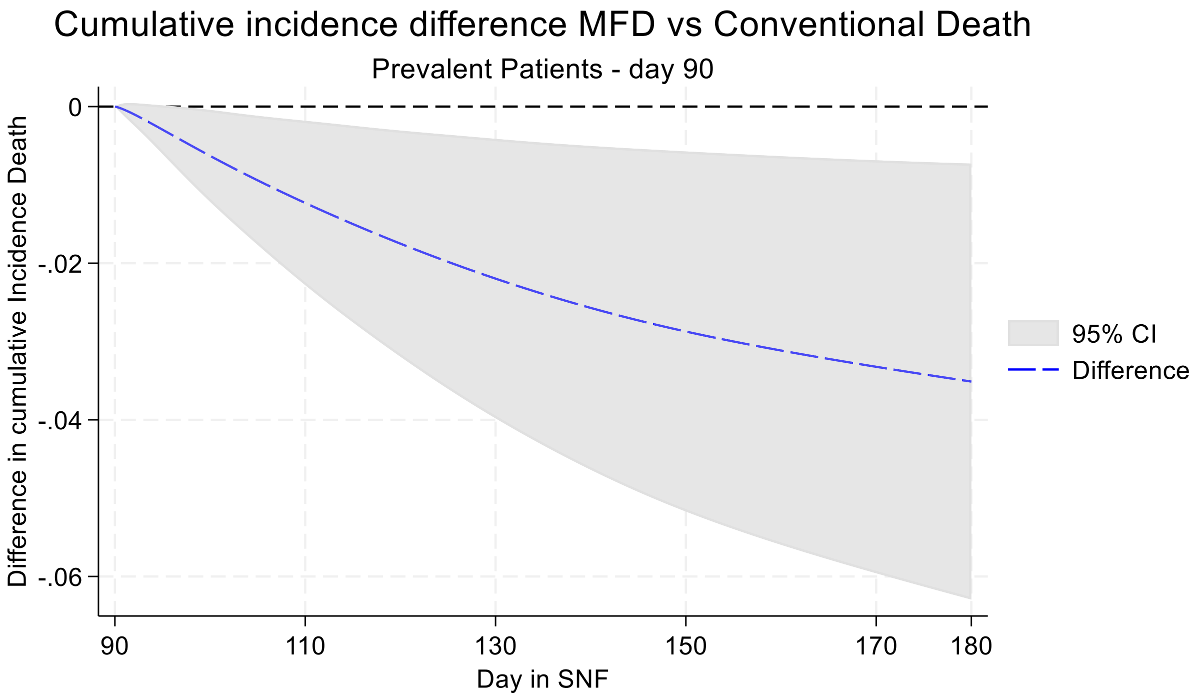

Figure s5. Dialytic Episodes Competing Risk Cumulative Incidence difference Death, MFD vs Conventional in adjusted model evaluated at median covariate values (Prevalent patients Day 90)

Observation/Impression days 90-180

Statistical significance as demonstrated by confidence interval excluding zero (horizontal line at zero) is achieved by day 150 in discharge to home but never in hospitalization. Death does avoid zero but is of small magnitude.

180-270 Days

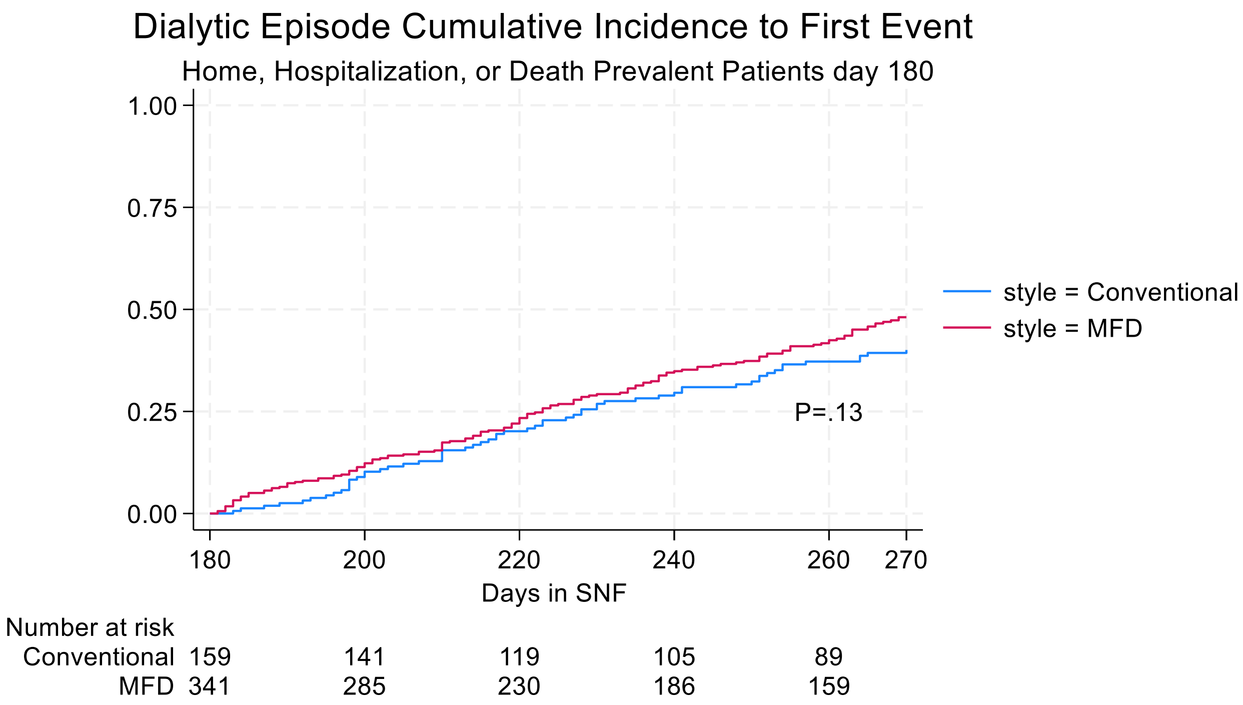

Figure s6. Dialytic Episodes with available covariates Cumulative Incidence to First Event (all cause: discharge home, hospital, or death) (Prevalent Patients Day 180)

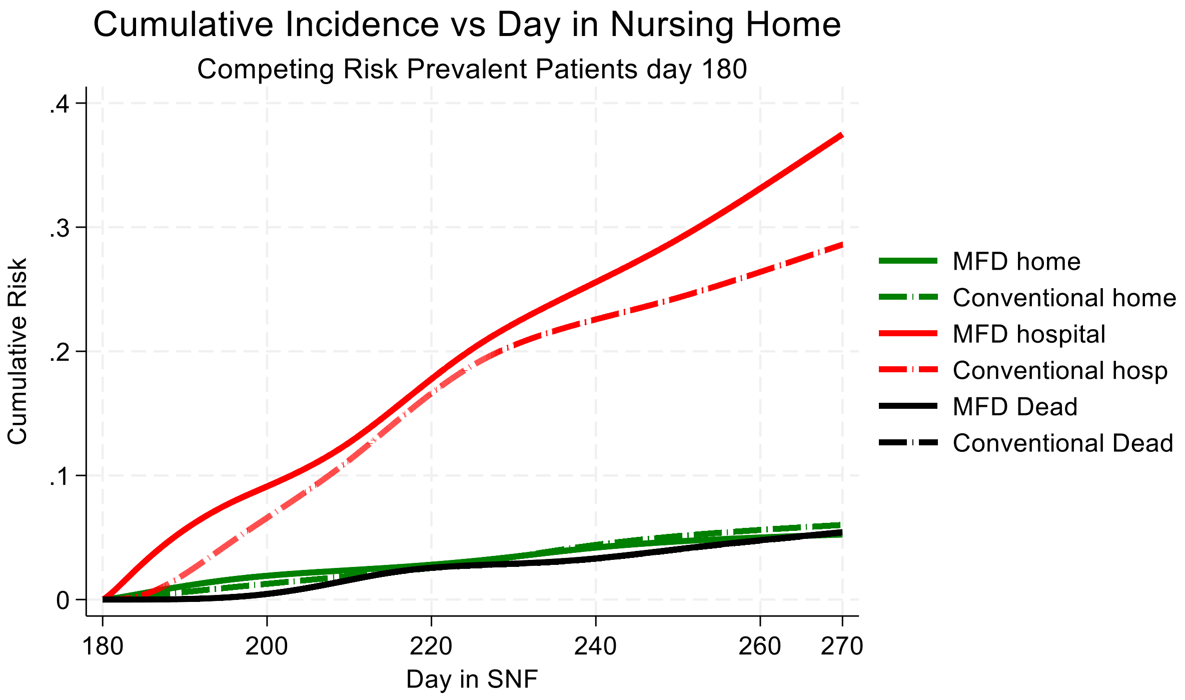

Figure s7. Dialytic Episodes Competing Risk Cumulative incidence home, hospital, death, MFD vs Conventional in adjusted model evaluated at median covariate values (Prevalent patients Day 180)

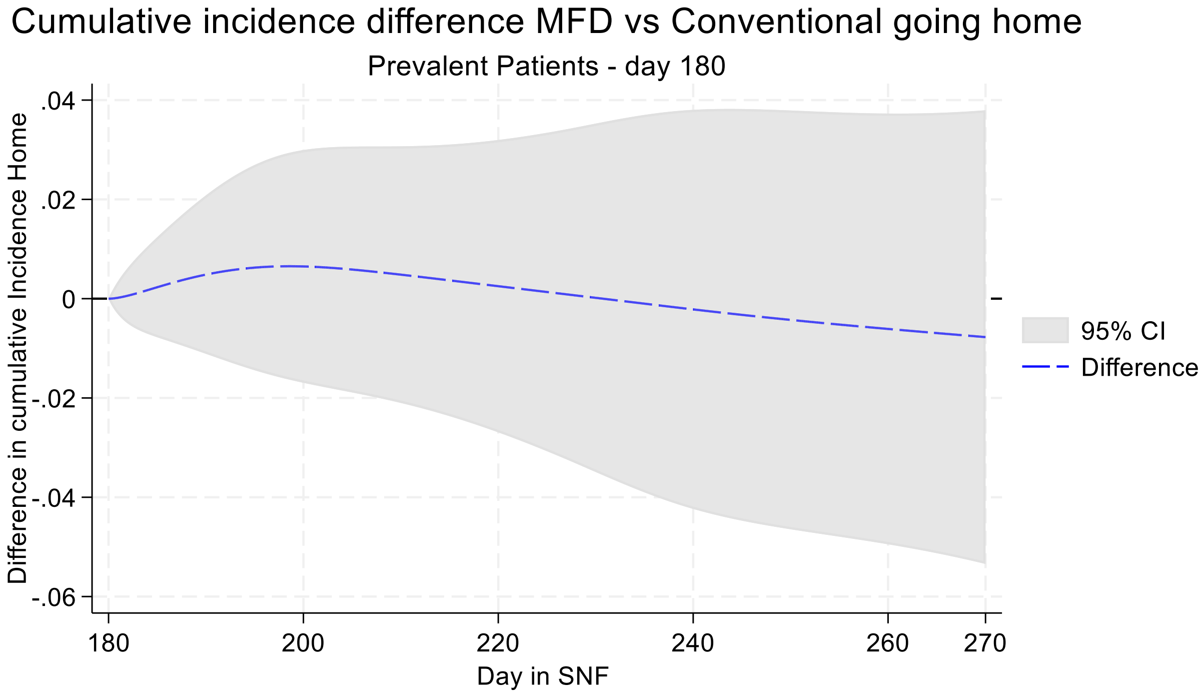

Figure s8. Dialytic Episodes Competing Risk Cumulative Incidence difference discharged home, MFD vs Conventional in adjusted model evaluated at median covariate values (Prevalent patients Day 180)

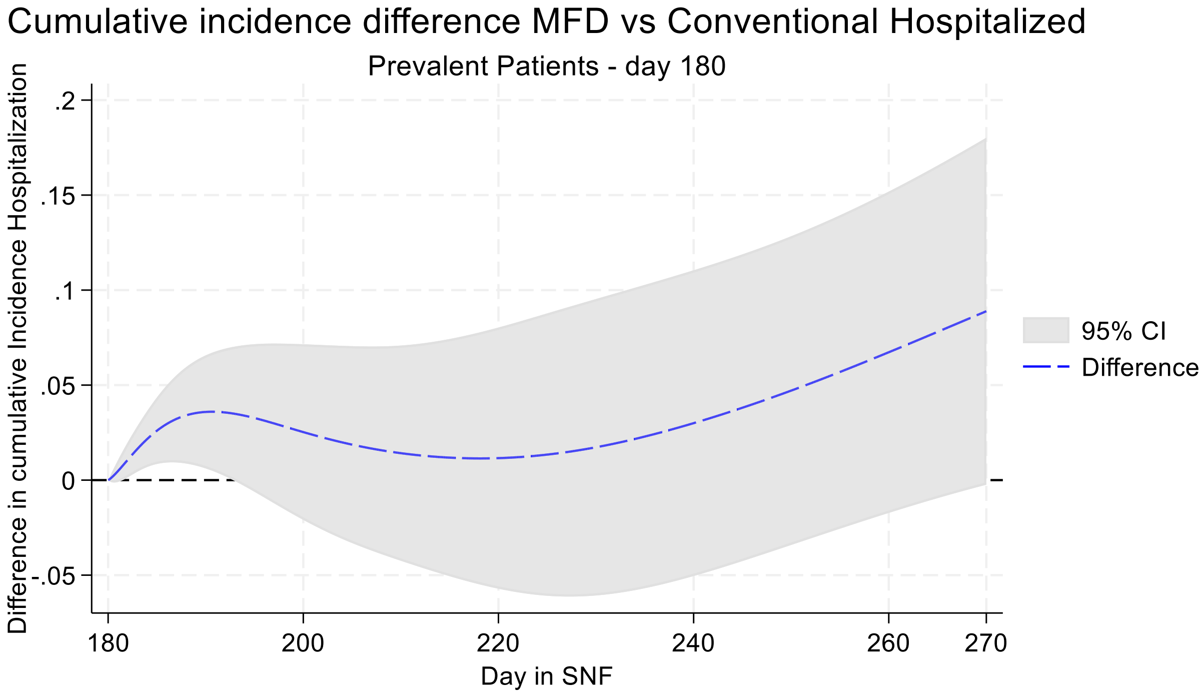

Figure s9. Dialytic Episodes Competing Risk Cumulative Incidence difference Hospitalized, MFD vs Conventional in adjusted model evaluated at median covariate values (Prevalent patients Day 180)

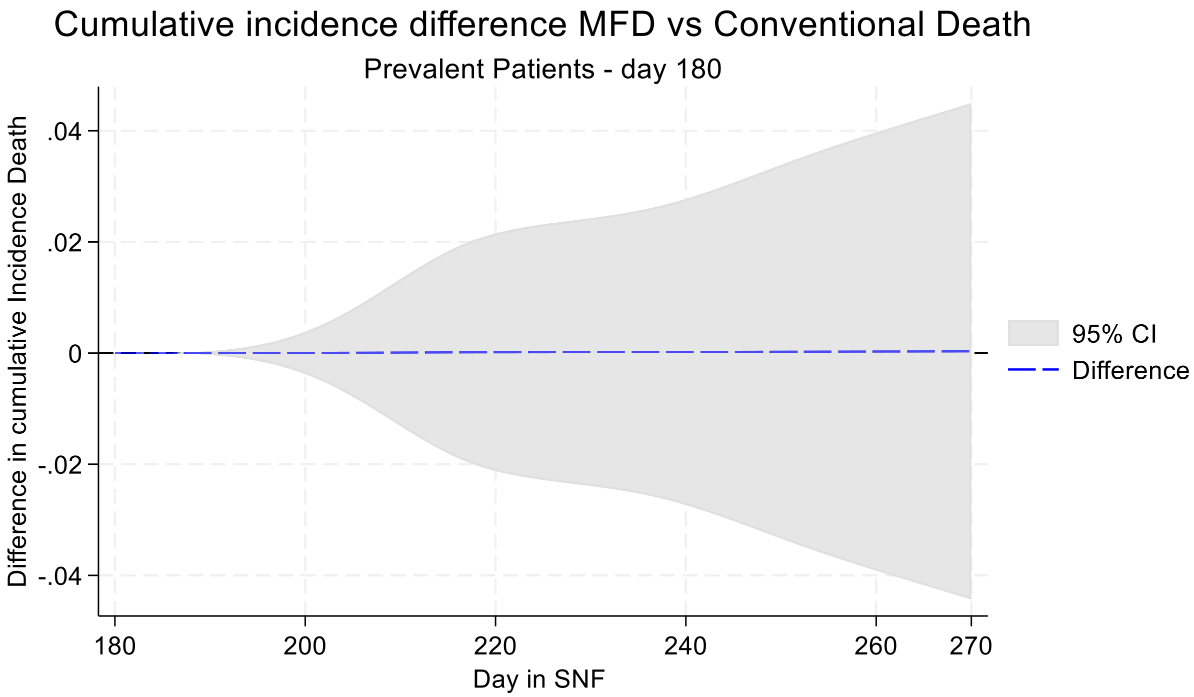

Figure s10. Dialytic Episodes Competing Risk Cumulative Incidence difference Death, MFD vs Conventional in adjusted model evaluated at median covariate values (Prevalent patients Day 180)

Observation/Impression days 280-270

No statistically significant difference MFD and Conventional in any of the three competing risks.

270-360 can not be done with spline modeling

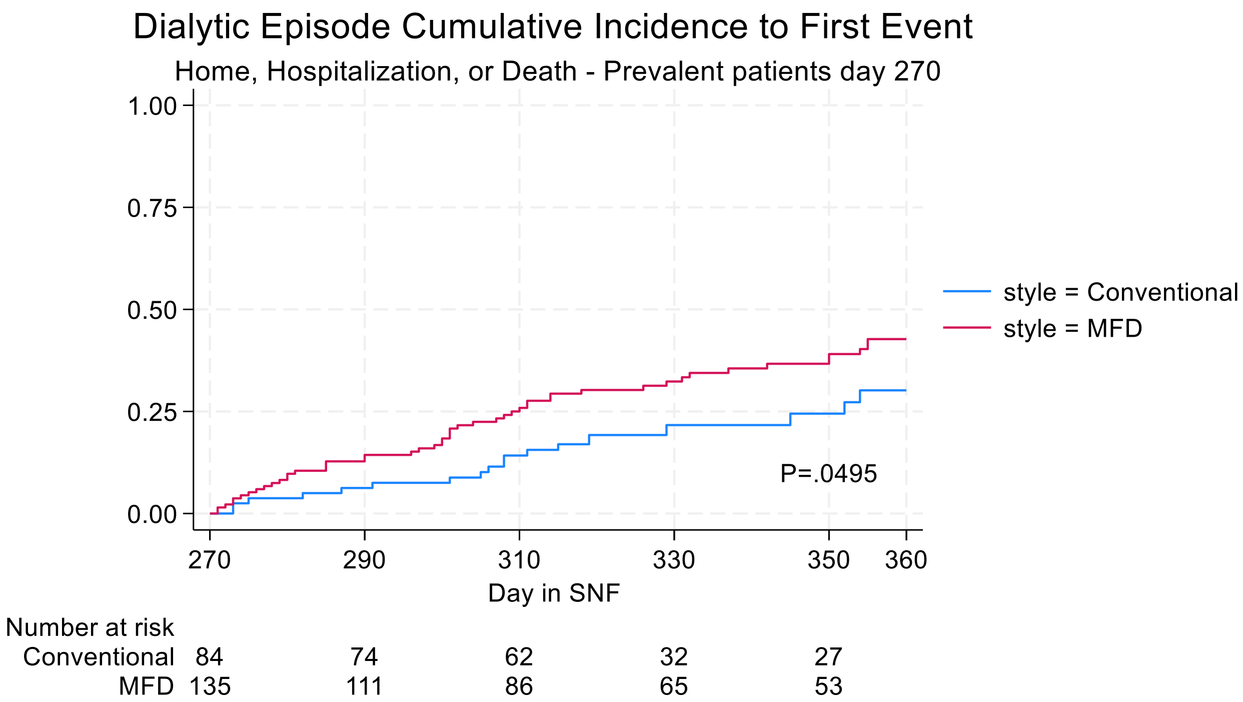

Figure s11. Dialytic Episodes with available covariates Cumulative Incidence to First Event (all cause: discharge home, hospital, or death) (Prevalent Patients Day 270)

180-360 Days

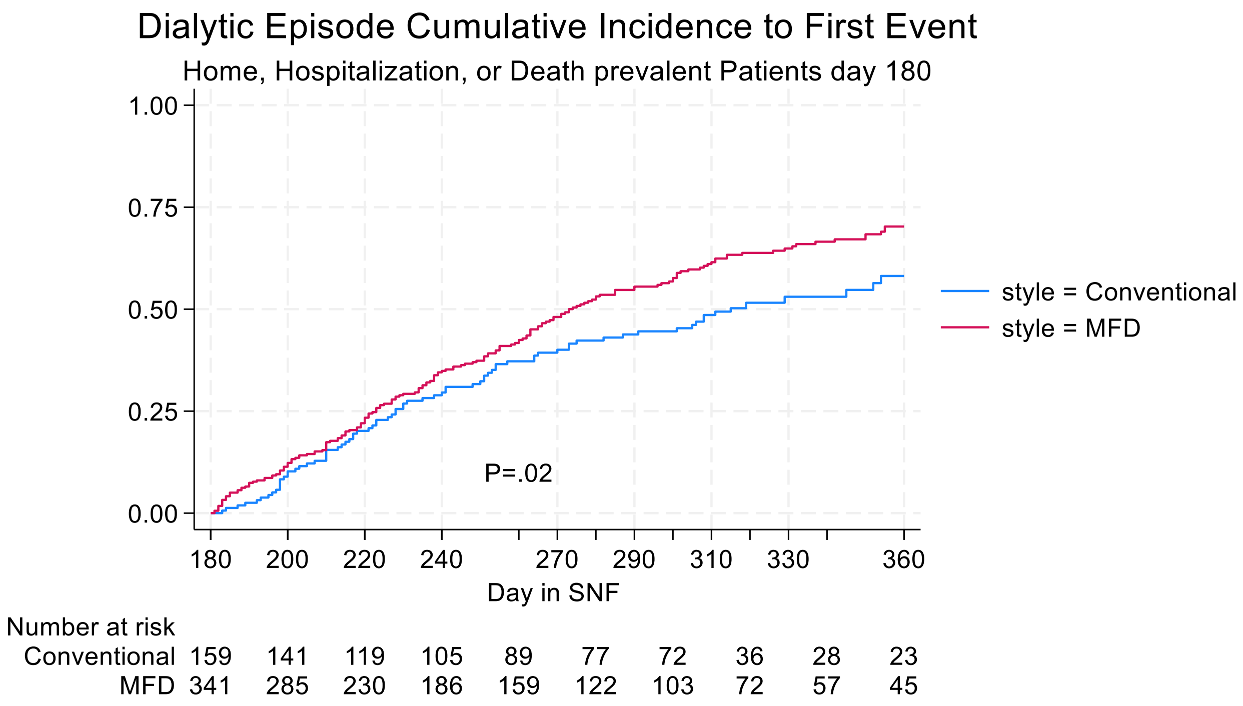

Figure s12. Dialytic Episodes with available covariates Cumulative Incidence to First Event (all cause: discharge home, hospital, or death) (Prevalent Patients Day 180) followed through day 360.

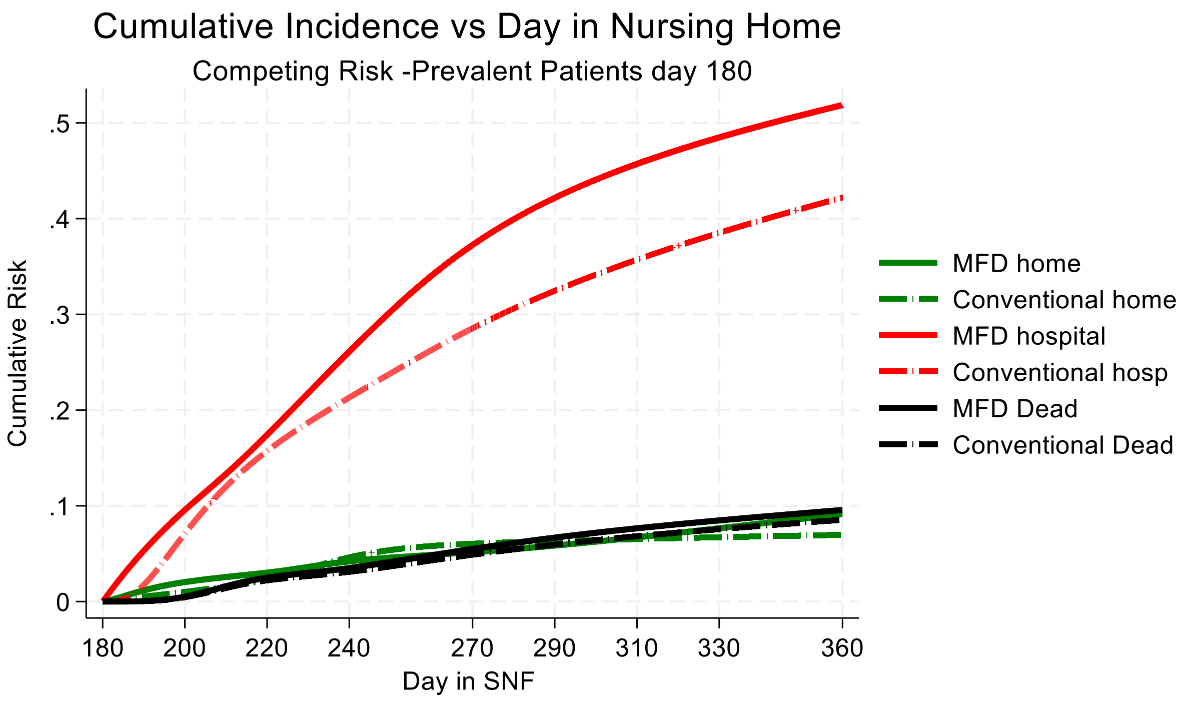

Figure s13. Dialytic Episodes Competing Risk Cumulative incidence home, hospital, death, MFD vs Conventional in adjusted model evaluated at median covariate values (Prevalent patients Day 180) followed through day 360

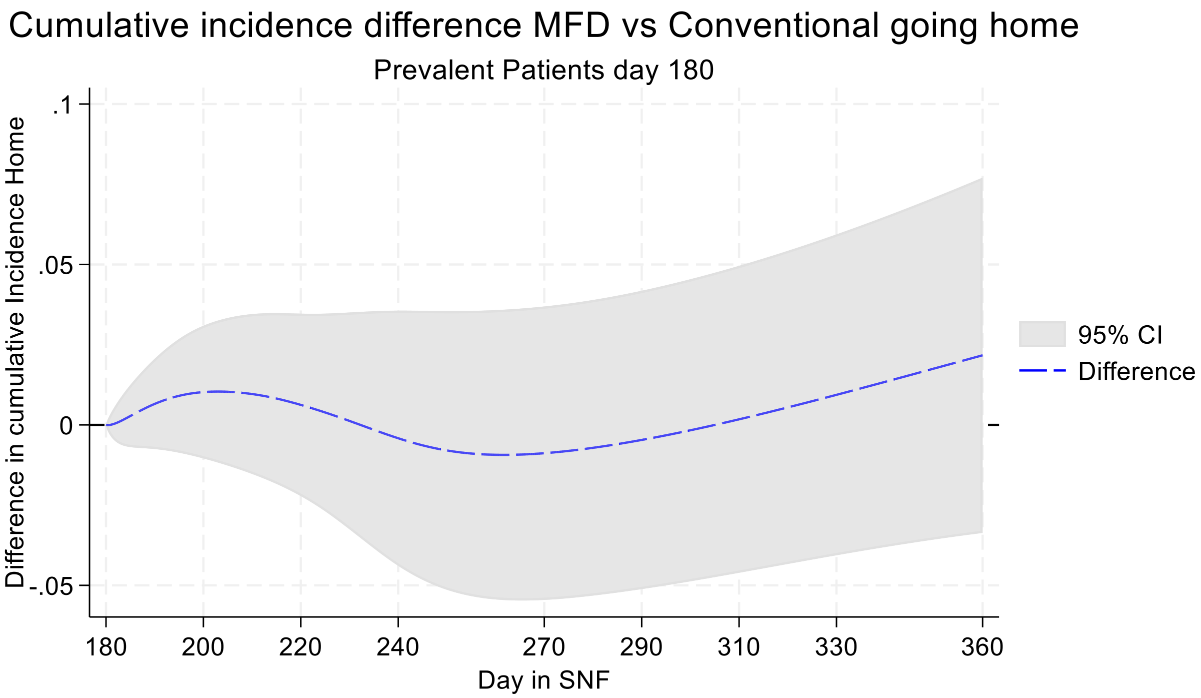

Figure s14. Dialytic Episodes Competing Risk Cumulative Incidence difference discharged home, MFD vs Conventional in adjusted model evaluated at median covariate values (Prevalent patients Day 180) followed through day 360

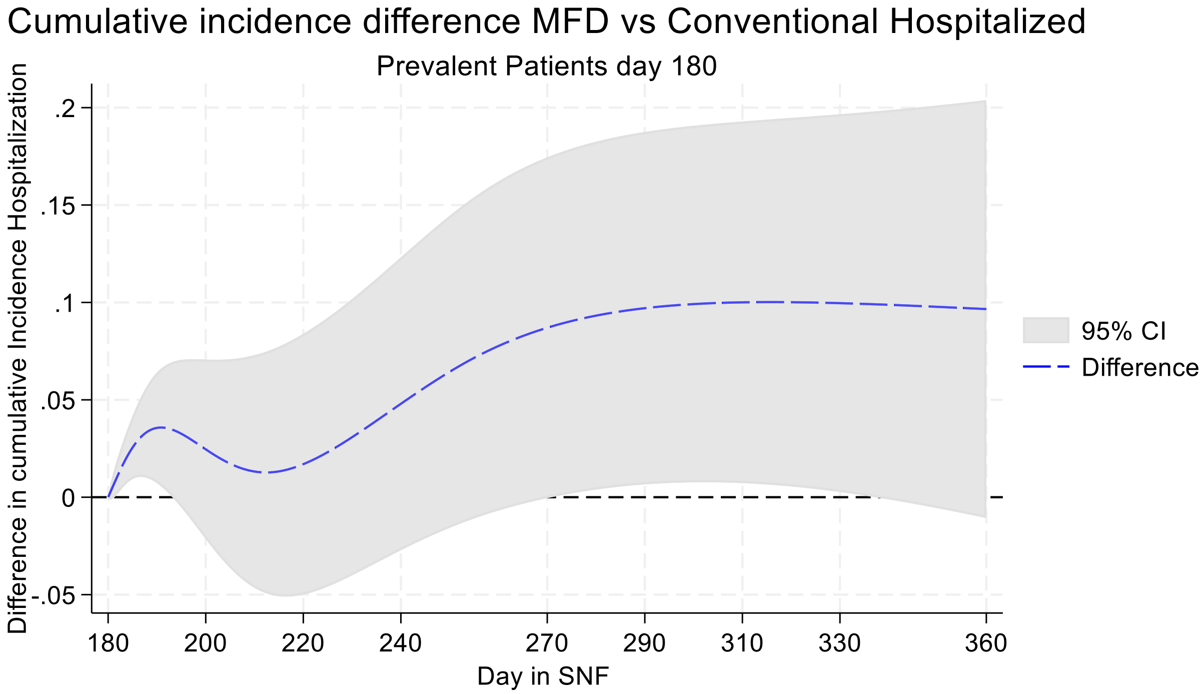

Figure s15. Dialytic Episodes Competing Risk Cumulative Incidence difference Hospitalized, MFD vs Conventional in adjusted model evaluated at median covariate values (Prevalent patients Day 180) followed through day 360

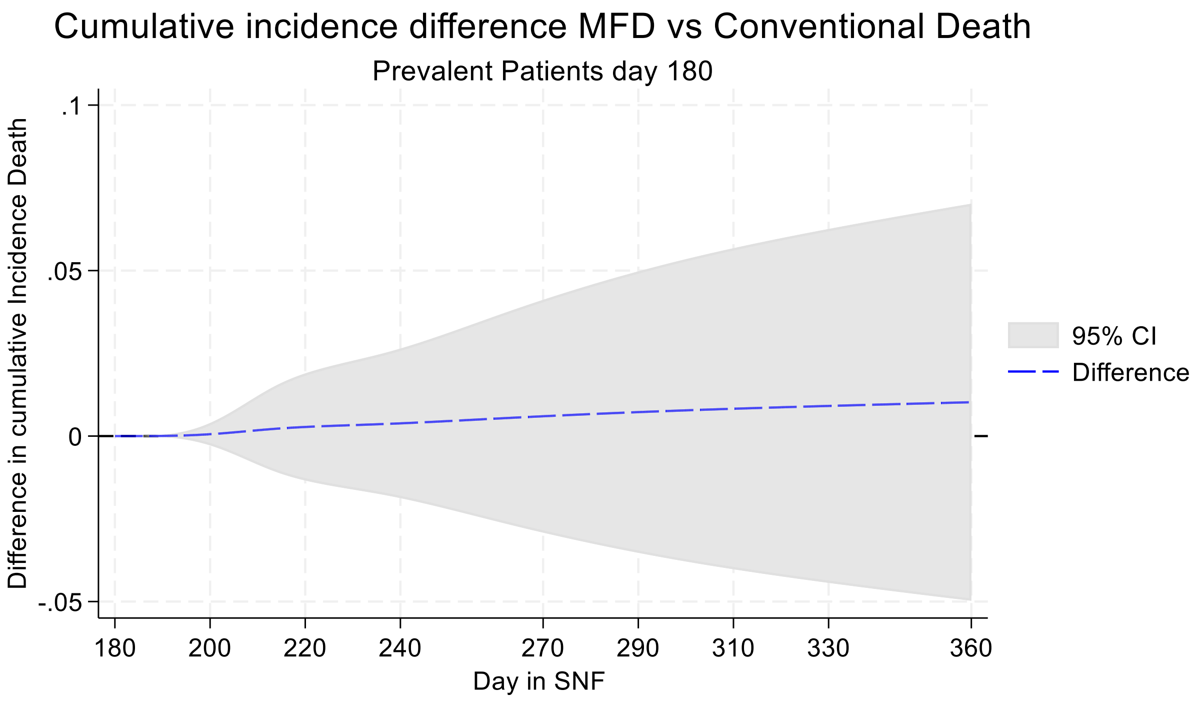

Figure s16. Dialytic Episodes Competing Risk Cumulative Incidence difference Death, MFD vs Conventional in adjusted model evaluated at median covariate values (Prevalent patients Day 180) followed through day 360

Observation/Impression days 180-365

All cause time to first discharge is statistically greater in MFD than conventional but does not show significance in modeling discharge to home or death. Hospitalization transiently achieves significance but both its transience and its magnitude make it less than fully convincing but worthy of further study.
